# Genome Sequence of a Candidate World Health Organization Reference Strain of Zika Virus for Nucleic Acid Testing

**DOI:** 10.1128/genomeA.00917-16

**Published:** 2016-09-01

**Authors:** Jan-Hendrik Trösemeier, Didier Musso, Johannes Blümel, Julien Thézé, Oliver G. Pybus, Sally A. Baylis

**Affiliations:** a Paul-Ehrlich-Institut, Langen, Germany; b Molecular Bioinformatics, Johann Wolfgang Goethe University, Frankfurt am Main, Germany; c Institut Louis Malardé, Tahiti, French Polynesia; d Department of Zoology, University of Oxford, Oxford, United Kingdom

## Abstract

We report here the sequence of a candidate reference strain of Zika virus (ZIKV) developed on behalf of the World Health Organization (WHO). The ZIKV reference strain is intended for use in nucleic acid amplification (NAT)-based assays for the detection and quantification of ZIKV RNA.

## GENOME ANNOUNCEMENT

The mosquito-borne Zika virus (ZIKV) is a member of the *Flavivirus* genus in the *Flaviviridae* family, and nearly 70 years after its discovery in Uganda, it is spreading widely in South and Central America, the Caribbean, and the Pacific. Infections are frequently asymptomatic or cause mild disease; however, severe complications have recently come to light, including central nervous system abnormalities (especially microcephaly) in fetuses/neonates and Guillain-Barré syndromes in adults. ZIKV is transmitted by the bite of infected mosquitoes, but nonvector-borne transmission has been reported, through materno/fetal, sexual, and blood transfusion (1–3). Diagnosis of acute ZIKV infection relies upon the detection of virus RNA in plasma, urine, and other body fluids (2, 4). In order to implement robust diagnostic testing methods, a candidate World Health Organization (WHO) reference material (international standard), intended to be used for the quantitation of ZIKV RNA and evaluated in an international study, was developed. The intended WHO ZIKV reference material is similar to those developed for other blood-borne viruses (5).

The ZIKV strain PF13/251013-18 was isolated from serum specimen from a French Polynesian patient in 2013 (6). ZIKV RNA was extracted from Vero E6 cell culture supernatants, from low-passage virus, using the QIAamp MinElute virus spin kit (Qiagen GmbH, Hilden, Germany). The passage number was identical to the virus stock used in the preparation of the lyophilized candidate reference material. Sequencing was performed using Illumina MiSeq (2 × 300 bp).

Sequencing reads were preprocessed by quality filtering with PRINSEQ (7). *De novo* assembly was performed with the MIRA 4 assembler (8). On the basis of BLAST searching against the NCBI database, the sequence with the highest identity and *E* score was chosen for reference-assisted scaffolding using Mira and BWA (9). Consensus sequences, based on reference-assisted scaffolding, were constructed using SAMtools (10). The final ZIKV sequence has a length of 10,769 bp.

The ZIKV strain PF13/251013-18 genome sequence was aligned along with all published complete and partial (>80% coverage) ZIKV genomes belonging to the Asian genotype using MAFFT (11). A maximum-likelihood phylogenetic tree was inferred using PhyML (12) under a reversible nucleotide substitution model with a proportion of invariant sites (GTR + I), which was the best-fitting model, as determined by jModelTest (13). Statistical support for phylogenetic nodes was assessed using a bootstrap approach (with 100 replicates). The resulting ZIKV Asian genotype phylogeny is in accordance with previous studies (14). ZIKV strain PF13/251013-18 is an outgroup to the American clade (bootstrap support, 77%) and is genetically similar to another genome from the French Polynesia outbreak (isolate H/PF/2013).

The demonstration that the WHO ZIKV reference strain is closely related to currently circulating viruses in the Pacific, Central and South America, and Caribbean is important, because the selection of viruses for the development of WHO reference materials has been based on strains representative of viruses that are clinically significant and have widespread distribution. Knowledge of the sequence of the PF13/251013-18 ZIKV isolate is essential for oligonucleotide design for NAT assays to ensure adequate detection and quantification of the WHO reference material.

### Accession number(s).

The sequence of the candidate WHO reference strain PF13/251013-18 has been deposited in GenBank under accession no. KX369547.
